# Factors associated with cellulitis in lymphoedema of the arm – an international cross-sectional study (LIMPRINT)

**DOI:** 10.1186/s12879-023-08839-z

**Published:** 2024-01-18

**Authors:** Ewa Anna Burian, Peter J. Franks, Pinar Borman, Isabelle Quéré, Tonny Karlsmark, Vaughan Keeley, Junko Sugama, Marina Cestari, Christine J. Moffatt

**Affiliations:** 1 Department of Dermato-Venereology & Wound Healing Centre, Bispebjerg Hospital, Bispebjerg Bakke 23, Copenhagen, 2400 Denmark; 2 Centre for Research and Implementation of Clinical Practice, London, UK; 3 Medical Faculty, Department of Physical Medicine and Rehabilitation, Ankara Medipol University, Ankara, Turkey; 4 CHU Montpellier, University of Montpellier, IDESP, Montpellier, France; 5 Lymphoedema Department, University Hospitals of Derby and Burton NHS Trust, Derby and University of Nottingham Medical School, Nottingham, UK; 6 Research Centre for Implementation Nursing Science Initiative, Research Promotion Headquarters, Fujita Health University, Aichi, Japan; 7 Pianeta Linfedema Study Centre, Terni, Italy; 8 Nottingham University Hospitals NHS Trust, Nottingham, UK

**Keywords:** Cellulitis, Erysipelas, Lymphedema, Lymphoedema, Chronic edema, Breast-neoplasm, Breast cancer

## Abstract

**Background:**

Lymphoedema is a globally neglected health care problem and a common complication following breast cancer treatment. Lymphoedema is a well-known predisposing factor for cellulitis, but few have investigated the risk factors for cellulitis in this patient cohort on an international level. The aim of this study was to identify the frequency of cellulitis in patients with lymphoedema of the arm, including potential risk factors for cellulitis.

**Methods:**

An international, multi-centre, cross-sectional study including patients with clinically assessed arm lymphoedema. The primary outcome was the incidence of cellulitis located to the arm with lymphoedema within the last 12 months, and its potential associated risk factors. The secondary outcome was life-time prevalence of cellulitis. Adults with clinically-assessed arm lymphoedema/chronic oedema (all causes) and able to give informed consent were included. End-of-life-patients or those judged as not in the patient’s best interest were excluded. Both univariable and multivariable analysis were performed.

**Results:**

A total of 2160 patients were included from Australia, Denmark, France, Ireland, Italy, Japan, Turkey and United Kingdom. Secondary lymphoedema was present in 98% of the patients; 95% of these were judged as related to cancer or its treatment. The lifetime prevalence of cellulitis was 22% and 1-year incidence 11%. Following multivariable analysis, factors associated with recent cellulitis were longer swelling duration and having poorly controlled lymphoedema. Compared to having lymphoedema less than 1 year, the risk increased with duration: 1–2 years (OR 2.15), 2–5 years (OR 2.86), 5–10 years (OR 3.15). Patients with well-controlled lymphoedema had a 46% lower risk of cellulitis (OR 0.54, 95% CI 0.39–0.73, *p* < 0.001). More advanced stages of lymphoedema were associated with cellulitis even after adjustment for swelling duration and control of swelling by logistic regression (stage II OR 5.44, stage III OR 9.13, *p* = 0.002), demonstrated in a subgroup analysis.

**Conclusion:**

Patients with advanced arm lymphoedema are at particular risk of developing cellulitis. Prevention of lymphoedema progression is crucial. The results lend towards a positive effect of having well-treated lymphoedema on the frequency of cellulitis.

**Supplementary Information:**

The online version contains supplementary material available at 10.1186/s12879-023-08839-z.

## Background

One in five women develop lymphoedema following breast cancer treatment [1]. This serious complication is caused by damage to the lymphatics, often through surgery or radiation [1, 2]. The extent of the surgery determines the risk for lymphoedema, being four times increased after axillary lymph node dissection (20%) compared to sentinel node biopsy (6%) [1]. Although lymphoedema of the arm can arise due to congenital lymphatic malformations, as in primary lymphoedema, secondary causes due to cancer or its treatment are much more common. Factors that may influence the development of swelling in cancer-related lymphoedema include obesity, cancer related-drugs [3] and genetic background [4].

In lymphoedema, protein rich lymph accumulates in the interstitial space. With time excessive fat tissue and fibrosis may develop. Consequently, the arm swells, which has a substantial impact on patients’ quality of life [5]. Recurrent episodes of cellulitis, synonymously often called erysipelas, is a significant complication. Cellulitis is a bacterial skin infection, caused by *Streptococcus pyogenes* and/or less frequently *Staphylococcus aureus* [6]. Cellulitis is one of the most frequent causes of emergency admissions [7] with an overall mortality of 2.5-5% [8, 9]. Recently an annual rising incidence of cellulitis around 5% was reported [10].

Damaged lymphatics, as seen in lymphoedema, is a well-established risk factor for cellulitis in breast cancer survivors [11–14], potentially due to a locally impaired immunity. Infections however, also damage the lymphatics. MRSA-infected mice have decreased lymphatic transportation over six months following bacterial clearance, due to the release of toxins causing lymphatic muscle cell death [15]. Similar findings are reported in humans, where breast cancer patients with previous cellulitis have more extensive lymphatic injury and leakage [12, 16]. Hence, lymphoedema predisposes to cellulitis, and cellulitis predisposes to lymphoedema [16–18].

Previous studies have focused on the epidemiology of cellulitis of the legs. Important risk factors identified in a meta-analysis, included previous cellulitis, wounds, leg ulcers, excoriating skin diseases and lymphoedema [19]. Similar results in breast cancer-related lymphoedema (BRCL) are reported with an increased risk of cellulitis (OR 9.6. 95% CI 1.2–79.8) [20]. Despite the serious consequences of lymphoedema, it has historically been subject to scientific neglect [3]. Few have attempted to depict risk factors of cellulitis in patients with lymphoedema of the arm across an international cohort.

The objective of this study was to determine the 1-year incidence and life-time prevalence of cellulitis in patients with clinically assessed lymphoedema of the arm (oedema > 3 months), and to determine factors associated with a recent case of cellulitis.

## Materials and methods

### Study design

A prospective, international, multi-centre, cross-sectional sub-study of the main project LIMPRINT^1^; a project launched to investigate the epidemiology and consequences of chronic oedema/lymphoedema. In this study, cellulitis located to the arm in patients with arm lymphoedema were investigated. Patients were recruited from both in- and outpatient facilities. The necessary approvals from the Ethical Review Committee including research and service development committees were collected by each country and centre. The methodology used in LIMPRNT and its validation has previously been published elsewhere [21].

### Primary outcome

The incidence of cellulitis located in the arm with lymphoedema within the last 12 months, and its potential associated risk factors.

### Eligibility criteria

Adults over 18 years of age, with clinically-assessed arm lymphoedema/chronic oedema (all causes included) and able to give informed consent. End-of-life-patients or those judged as not in the patient’s best interest by the investigator were excluded. In order to be assured that cellulitis was associated with the arm and related structures patients were excluded if they had swelling of other sites except for swelling of the back, chest and breast (“midline”).

### Study definitions

#### Lymphoedema/chronic oedema

Lymphoedema is caused by impaired lymphatic transport. Yet, swelling is often complex with several contributing factors. The term “chronic oedema” has therefore been introduced; defined as oedema > 3 months, regardless of the cause(s) [22]. The prominent role of the lymphatics for drainage in all chronic oedema has been recognized [3]. Patients with swelling of the arm > 3 months, judged by a positive Pitting Oedema Test and/or a positive Stemmer’s sign (a skin fold cannot be pinched at the base of the second finger) were included [23]. Throughout this manuscript, we use the term lymphoedema, as it is the most used description in the literature.

#### Cellulitis

An acute onset of red, warm, swollen and painful skin, often accompanied by fever and a rapid response to antibiotics. Cellulitis were confirmed by interview with the patient and/or review of the medical records by trained investigators, and if possible a physical examination. Erysipelas and cellulitis was regarded as a spectrum of the same infectious skin disease [6].

### ISL classification

The severity of lymphoedema was determined using the ISL classification (International Society of Lymphology scale) [24], including:

#### Stage I

Accumulation of fluid which subsides with limb elevation. Pitting may occur.

#### Stage II

Limb elevation alone rarely reduces swelling, and pitting is manifest. Later in this stage the limb may not pit as subcutaneous fat and fibrosis develop.

#### Stage III

Pitting is absent. Fat, fibrosis, and warty overgrowths have developed.

### Data collection

Data were collected by trained health care professionals using a standardized core tool. This check-off scheme was used in all patients, and included a questionnaire and a physical examination describing e.g. demographics, comorbidities, lymphoedema duration, lymphoedema treatment, site(s) of swelling, presence of wounds etc. Lymphoedema specialists confirmed the underlying lymphoedema classification, and the diagnosis of cellulitis. Some centres with the appropriate expertise, undertook the staging procedure of lymphoedema (ISL), as described above. The cancer tool was a questionnaire used at some sites, in patients with lymphoedema due to cancer and/or it’s treatment. The tool included domains to subclassify the type of cancer, including treatments used as surgery, radiation therapy or chemotherapy. A further description of the tools has been published (open access) [21].

### Variables

Site of lymphoedema was collected using a body map, and was classified as either primary (congenital) or secondary (acquired). Secondary lymphoedema was further classified as:

1. *Cancer-related lymphoedema*: lymphoedema judged to be caused by cancer/metastatic disease and/or by cancer treatment (as axillary lymph node dissection), or.
2. *Non-cancer-related lymphoedema*: lymphoedema due to venous disease, obesity, immobility, lymphatic filariasis and/or “other”.

Variables tested for an association with a current episode of cellulitis (< 12 months) were: sex, age, weight category, concomitant diseases, presence of a wound, classification of chronic oedema (primary vs. secondary), etiology of chronic oedema, ISL stage, duration of leg oedema, concomitant midline swelling, mobility, control of swelling, pitting oedema, tissue quality, and Stemmers sign. Weight categories was assessed by estimating the body mass index (BMI) as either underweight (BMI < 20; not included in this analysis), normal weight^2^ (BMI 20–30), obesity (BMI 30–40) or morbid obesity (BMI > 40). Control of swelling was a subjective evaluation based on a clinical examination made by trained investigators, and was judged as either present, absent or “don’t know”. When in doubt the investigator was instructed to contact the lead clinician for clarity on the chronic oedema status.

### Statistics

Due to the explorative study design, a sample size determination was not performed. The principal analysis was undertaken comparing clinical variables with cellulitis within the past 12 months as the dependent variable. Both univariable and multivariable analysis were performed. Determination of the independent factors within a multivariable model were undertaken using the logistic model with a stepwise elimination until all remaining variables had a *p* < 0.05. An additional analysis examining the severity of lymphoedema in a subgroup of patients was performed. Data is presented as proportional OR with 95% confidence intervals (CI). Missing data were not imputed and therefore remained missing. Stata 12 (Statacorp, Texas) was used for statistical analyses.

## Results

### Study sites and population

Between June 2014-August 2017, 2160 patients with lymphoedema of the arm were recruited from 27 centres in eight countries: Australia, Denmark, France, Ireland, Italy, Japan, Turkey and United Kingdom. The majority of patients were recruited from specialist lymphoedema services (89%), the rest being identified in hospitals (11%) and community care/others (5 patients).

### Demographics

The majority were women (2063/2160 = 96%). Primary lymphoedema was present in 2% of the patients (48/2155), while the rest had secondary lymphoedema. Out of those with secondary lymphoedema, 95% were related to cancer or its treatment (2000/2099), the rest being due to trauma/surgical procedures, venous disorders/thrombotic, immobility, skin disorders including cellulitis, multiple contributing factors or other/unknown. The cancer questionnaire was completed in 457 patients. The most common cancer diagnosis was breast cancer (450/457 = 98%), of which the majority received axillary node clearance (435/438 = 99%), radiation (366/368 = 99%) and/or chemotherapy (394/399 = 99%). In the total cohort, the duration of lymphoedema was of over one year in 76% of cases (1643/2158). Reduced mobility of the arm was seen in 27% (573/2159), and one third had concomitant swelling of the upper torso. Compression therapy was used in 83% of the patients (1782/2158). Good control of swelling was judged in 74% (1364/1841). Patient characteristics, cancer types and treatment modalities are presented in Table 1, and Appendices Table A1 and A2.

**Table 1:** Demographics of patients with lymphoedema of the arm (n = 2160)

Characteristic(s)	Number of patients (%)
Clinical area	
Community care	1 (0.05)
Hospital services	238 (11.02)
Lymphoedema specialist	1917 (88.75)
Other	4 (0.19)
missing	0
Age, mean (sd)	60.79 (12.89)
missing	0
Female	2063 (95.51)
missing	0
Weight category	
Underweight	23 (1.07)
Normal weight	1430 (66.30)
Obesity	635 (29.44)
Morbid obesity	69 (3.20)
missing	3
Concomitant disease	
Diabetes	210 (9.72)
missing	0
Heart failure/ ischemic heart disease	102 (4.72)
missing	0
Neurological disease	52 (2.41)
missing	3
Classification of lymphoedema	
Primary	48 (2.23)
Secondary (all causes)	2107 (97.77)
missing	5
Non-cancer secondary causes of lymphoedema	99 (4.71)
Trauma or surgical procedures	25
Venous disorder incl. thrombotic	10
Immobility	8
Skin disorders incl. cellulitis	11
Obesity	1
Multiple contributing factors	10
Other or unknown	29
missing	5
Cancer-related secondary lymphoedema	2000 (95.28)
missing	8
Cancer treatment	1947 (97.50)
missing	3
Metastatic disease	72 (3.61)
missing	3
Cancer type	
Breast	450
Bladder	7
Melanoma	4
Endometrial	1
Head and neck	1
Ovarian	1
Other	3
missing	0
Duration of arm lymphoedema	
< 1 year	515 (23.86)
1–2 years	349 (16.17)
2–5 years	557 (25.81)
> 5–10 years	445 (20.62)
> 10 years	292 (13.53)
missing	2
General Mobility	
Normal	1992 (92.31)
Walking aid	136 (6.30)
Chair/ bed bound	30 (1.39)
missing	2
Arm mobility	
Full	1586 (73.49)
Limited/ none	573 (26.51)
missing	2
Lifetime cellulitis	483 (22.36)
missing	0
Recent cellulitis (< 12 months)	243 (11.25)
missing	0
Upper torso swelling	679 (31.44)
missing	0
Presence of an arm wound	15 (0.70)
missing	4
Treatment with compression therapy	
Compression garment	1731 (80.32)
Multilayer bandage	725 (33.46)
Compression wrap	114 (5.27)
At least one of the above	1782 (82.58)
No compression	376 (17.42)
missing	2
Good control of lymphoedema (well-controlled swelling)	1364 (74.09)
missing	319

### Frequency of cellulitis

The lifetime prevalence of cellulitis was 22% (483/2160), and of these 242 (11%) experienced cellulitis within the last 12 months. The frequency across countries is depicted in Table 2. Among the patients completing the cancer tool 14% had cellulitis within the last year (62/457).

**Table 2:** History of arm cellulitis (< 12 months) in patients with lymphoedema by country

Country	Total number of patients with lymphoedema of the arm	History of cellulitis (< 12 months)	Percentage
Australia	1	0	0
Ireland	3	0	0
United Kingdom	991	91	9.18
Italy	545	58	10.64
Japan	27	3	11.11
Turkey	433	59	13.63
France	129	24	18.60
Denmark	31	7	22.58
**Total**	**2160**	**242**	**11.20**

### Factors associated with cellulitis: univariate analysis

Factors significantly associated with cellulitis within the last 12 months were: walking with aid (OR 0.35), neurological disease (OR X^3^), longer swelling duration (OR 2.03–2.87, duration > 1 year-10 years), treatment with compression therapy (OR 1.69) and having well-controlled lymphoedema (OR 0.62). There was no significant association to sex, age, weight category, arm mobility, diabetes, heart failure/ischemic heart disease, type of lymphoedema (primary vs. secondary), whether secondary lymphoedema was related to cancer or its treatment or not, concomitant upper torso swelling, or the presence of an arm wound. General factors investigated for an association are presented in Table 3, and for lymphoedema-related factors in Table 4.

**Table 3:** Explanatory variables for cellulitis in patients with lymphoedema of the arm by univariate analysis (n = 2160)

Risk factor	No cellulitis N (%)	Cellulitis N (%)	OR 95% CI	*P*-value
Sex				
Female	1830 (95.41)	233 (96.28)	1.00	
Male	88 (4.59)	9 (3.72)	0.80 (0.40, 1.62)	0.54
Age				
< 45 years	193 (10.07)	16 (6.64)	1.00	
45–64 years	977 (50.97)	126 (52.28)	1.56 (0.90, 2.68)	
65–74 years	432 (22.54)	65 (26.97)	1.81 (1.02, 3.22)	0.27
75–84 years	262 (13.67)	28 (11.62)	1.30 (0.68, 2.45)	
85 + years	53 (2.76)	6 (2.49)	1.37 (0.51, 3.66)	
Weight category				
Normal weight	1274 (66.49)	156 (64.73)	1.00	
Under weight	22 (1.15)	1 (0.41)	0.37 (0.05, 2.77)	
Obesity	559 (29.18)	76 (31.54)	1.11 (0.83, 1.49)	0.66
Morbid obesity	61 (3.18)	8 (3.32)	1.07 (0.50, 2.28)	
Leg mobility				
Walks unaided	1760 (91.81)	231 (95.85)	1.00	
Walks with aid	131 (6.83)	6 (2.49)	0.35 (0.15, 0.80)	0.03
Chair/bed bound	26 (1.36)	4 (1.66)	1.17 (0.41, 3.39)	
Arm mobility				
Full	1410 (73.55)	176 (73.03)	1.00	
Limited/ none	507 (26.45)	65 (27.69)	1.03 (0.76, 1.39)	0.86
Diabetes				
Absent	1730 (90.20)	220 (90.91)	1.00	
Present	188 (9.80)	23 (9.09)	0.92 (0.58, 1.46)	0.73
Heart failure/ ischemic heart disease				
Absent	1827 (95.26)	231 (95.45)	1.00	
Present	91 (4.74)	11 (4.55)	0.96 (0.50, 1.81)	0.89
Neurological disease				
Absent	1863 (97.28)	242 (100.00)		
Present	52 (2.72)	0 (0)*		0.009

*OR cannot be produced as there were no patients with neurological disease in the cellulitis group

**Table 4:** Explanatory variables for cellulitis related to characteristics of lymphoedema of the arm, by univariate analysis (n = 2160)

	No cellulitis N (%)	Cellulitis N (%)	OR 95% CI	*P*-value
Lymphoedema duration n = 2158				
< 1 year	485 (25.31)	30 (12.40)	1.00	
1–2 years	310 (16.18)	39 (16.12)	2.03 (1.24, 3.34)	
2–5 years	478 (24.95)	79 (32.64)	2.67 (1.72, 4.14)	< 0.001
5–10 years	378 (19.73)	67 (27.69)	2.87 (1.83, 4.50)	
> 10 years	265 (13.83)	27 (11.16)	1.65 (0.96, 2.83)	
Classification of lymphoedema n = 2155				
Primary	41 (2.14)	7 (2.89)	1.00	
Secondary	1872 (97.86)	235 (97.11)	0.74 (0.33, 1.66)	0.46
Secondary lymphoedema n = 2099				
Cancer-related	1778 (95.28)	222 (95.28)	1.00	
Non-cancer-related	88 (4.72)	11 (4.72)	1.00 (0.52, 1.90)	0.99
Cancer-related secondary lymphoedema n = 1997				
Cancer treatment				
Absent	45 (2.54)	5 (2.25)	1.00	
Present	1730 (97.46)	217 (97.75)	1.13 (0.44, 2.87)	0.80
Cancer metastatic				
Absent	1709 (96.28)	216 (97.30)	1.00	
Present	66 (3.72)	6 (2.70)	0.72 (0.31, 1.68)	0.44
Concomitant upper torso swelling				
Absent	1311 (68.35)	170 (70.25)	1.00	
Present	607 (31.65)	72 (29.75)	0.91 (0.68, 1.22)	0.55
Arm wound				
Absent	1902 (99.32)	239 (99.17)	1.00	
Present	13 (0.68)	2 (0.83)	1.22 (0.27, 5.46)	0.79
Treatment with compression n = 2159				
Absent	348 (18.16)	28 (11.59)	1.00	
Present	1568 (81.84)	214 (88.43)	1.69 (1.12, 2.56)	0.011
Control of lymphoedema n = 1841				
Not controlled	402 (24.71)	75 (35.05)	1.00	
Good control	1225 (75.29)	139 (64.95)	0.62 (0.46, 0.83)	0.001

### Factors associated with cellulitis: multivariable analysis

Remaining significant factors associated with cellulitis (< 12 months) following multivariable analysis included: longer swelling duration and having well-controlled lymphoedema, Table 5. Compared to having lymphoedema less than 1 year, the risk increased proportionally with time: 1–2 years (OR 2.15), 2–5 years (OR 2.86), 5–10 years (OR 3.15) (*p* < 0.001). Having lymphoedema > 10 years showed a tendency of an increased risk (OR 1.70), but did not reach statistical significance. Well-controlled lymphoedema was associated with a 46% lower risk of cellulitis (OR 0.54, 95% CI 0.39–0.73, *p* < 0.001).

**Table 5:** Logistic regression analysis: Independent risk factors associated with cellulitis of the arm in patients with lymphoedema (n = 1839)

	OR 95% CI	*P*-value
Lympoedema duration		
< 1 year	1.00	
1–2 years	2.15 (1.74, 3.72)	
2–5 years	2.86 (1.76, 4.65)	< 0.001
5–10 years	3.15 (1.92, 5.17)	
> 10 years	1.70 (0.93, 3.09)	
Control of lymphoedema		
Not controlled	1.00	
Good control	0.54 (0.39, 0.73)	< 0.001

### Lymphoedema-related factors associated with cellulitis

A subgroup of 460/2160 (21%) patients had a further characterization of their lymphoedema made using an additional lymphoedema questionnaire, Table 6. Cellulitis was associated with having fibrotic skin (OR 1.80), a positive Stemmer’s sign (OR 2.96), and advanced stages of lymphoedema (ISL stage II OR 5.79, stage III OR 10.24 compared to stage I), on univariate analysis. These factors remained significant after adjustment for swelling duration and control by logistic regression (stage II OR 5.44 and stage III OR 9.13, *p* = 0.002).

**Table 6:** Explanatory variables for cellulitis related to the severity of lymphoedema of the arm, a sub-group analysis (n = 460)

	No cellulitis N (%)	Cellulitis N (%)	OR 95%CI	*P*-value
Pitting (n = 459)				
Non pitting	232 (58.44)	29 (46.77)	1.00	
Pitting	165 (41.56)	33 (53.23)	1.60 (0.93, 2.74)	0.085
Tissue quality (n = 459)				
Soft	314 (79.09)	42 (67.74)	1.00	
Hard (fibrotic)	83 (20.91)	20 (32.26)	1.80 (1.00, 3.23)	0.046
Stemmer’s sign (n = 458)				
Negative	217 (54.80)	18 (29.03)	1.00	
Positive	179 (45.20)	44 (70.97)	2.96 (1.65, 5.31)	< 0.001
ISL scale* (n = 460)				
Stage I	139 (34.92)	5 (8.06)	1.00	
Stage II	240 (60.30)	50 (80.65)	5.79 (2.26, 14.87)	< 0.001
Stage III	19 (4.77)	7 (11.29)	10.24 (2.95, 35.53)	
ISL scale* after adjustment for lymphoedema duration and control by logistic regression (n = 326)				
Stage I			1.00	
Stage II			5.44 (1.59, 18.60)	0.002
Stage III			9.13 (1.99, 41.84)	

*ISL scale = International Society of Lymphology scale (assessment of severity of chronic edema/lymphedema). ISL stage I: Early onset of the condition, with an accumulation of tissue edema that decreases with limb elevation. The edema may be pitting at this stage. ISL stage II: Limb elevation alone rarely reduces swelling and pitting is manifested. ISL stage III: The tissue is fibrotic and pitting is absent. Skin changes such as thickening, hyperpigmentation, increased skin folds, fat deposits, and warty overgrowths develop

## Discussion

This study supports that cellulitis in lymphoedema of the arm is an international problem, with an overall 1-year incidence of 11% and life-time prevalence of 22%. Independently associated factors for cellulitis within the last twelve months was longer duration of lymphoedema, while patients with well-treated lymphoedema had their risk reduced by half (*p* < 0.001). Importantly, patients with more clinically advanced stages of lymphoedema were at particular increased risk of a recent skin infection.

Increased frequency of cellulitis in BRCL has been known for decades [11, 13, 20] with a prevalence ranging between 6 and 40% [2, 11–13, 25]. Previous epidemiological studies [11–14, 20, 25, 26] have been small, lacked an objective/standardized diagnosis, been single centre, national or had an unclear follow-up time. One retrospective study specifically investigated risk factors in arm lymphoedema, where lymphoedema-onset-to-first-consultation, age at lymphoedema onset and radiotherapy were independently associated with cellulitis. Axillary lymph node excision and chemotherapy were not [25]. Following breast cancer treatment: lymphoedema, duration of lymphoedema, radiotherapy and educational level have been reported as risk factors for cellulitis [20].

The importance of the lymphatics in the antimicrobial defense may explain the increased frequency of cellulitis in these patients. A normal lymphatic system is crucial for an effective adaptive immune response, in which antigen presenting cells reach the local draining lymph nodes [27, 28]. Recent research also show that the lymphatics are essential for the innate defense [29, 30]. Lymph nodes harbor and recruit macrophages and neutrophils, catching bacteria arriving with the lymph. Within one hour after a *Staphylococcus aureus* infection, neutrophils invade the lymph node preventing dissemination [30]. Some have suggested that bacterial presence in lymph nodes may be important in developing a strong immune response [28]. Reasons lymphoedema predispose to cellulitis may therefore include a locally decreased immunity (impaired antigen presentation in the lymph node/transport) [31], a moist bacteria-friendly environment, skin breakages and impaired bacterial clearance due to an increased diffusion barrier.

Our results support the importance of proper lymphoedema control – a 46% lower risk of cellulitis in well-treated lymphoedema within the last year. Lymphoedema treatment includes *complete decongestive therapy (CDT)* consisting of a combination of skin care, manual lymphatic drainage, exercise and compression [24]. Compression is crucial [32], improving lymphatic return and reducing capillary filtration [33]. The majority of patients in our study received compression (80%), being either garments, multilayer bandages or wraps. Surprisingly, this was associated with an increased OR for cellulitis on univariate, but not on multivariable analysis. This may suggest that compression was used in those with advanced lymphoedema and/or that the compression was not effective. Importantly, one third of patients had a midline swelling, which may challenge the effectiveness of CDT. The importance of proper compression is underscored in a recent well-designed RCT. A reduction in cellulitis recurrence was seen in patients with chronic oedema of the legs using compression stockings (hazard ratio 0.23, 95% CI 0.09–0.59, *p* = 0.002) [34], and was cost-effective [35]. Further support is seen in a large prospective cohort with arm or leg lymphoedema, where CDT decreased the incidence of cellulitis from 1.1 infections/patient/year to 0.65 [36]. Further research is needed to depict what element(s) of CDT that are effective for the prevention of arm cellulitis.

The risk of cellulitis increased with the duration, and importantly, the severity of lymphoedema where fat and fibrosis accumulates. Patients with fibrosis on palpation or a positive Stemmers sign were at significant risk. These clinical findings can easily be used bedside. Advanced stages of BRCL (assessed by lymphography) has been associated with more frequent cellulitis [37]. Increased lymphatic damage assessed by lymphangiography in breast cancer patients with cellulitis has also been reported. Cellulitis was associated with 20% points more excess fat compared to those without cellulitis [12]. Clearly, measures to prevent progression of lymphoedema are desired. Although best practice documents strongly support usage of compression throughout the disease course [24, 32], high quality evidence is needed to prove efficacy in lymphoedema of the arm.

Once fat is deposited in late-stage lymphoedema, it cannot be removed by conservative measures. *Liposuction*, combined with compression, may be effective for selected patients [38]. Lee et al. reported a reduction of cellulitis from 0.5 episodes/year to 0.06 (*p* < 0.001) after liposuction of post mastectomy lymphoedema in a prospective cohort study [39]. Similar results were published in a small retrospective study [40]. Liposuction with compression does not seem to further impair the lymphatics, assessed in a small study by indirect lymphoscintigrams. The beneficial effects may be attributed to decreased lymph formation [33], enhanced blood flow, and optimized wearing of compression garments [39]. Early studies using *vascularized lymph node transfer* may reduce the limb volume [41, 42] and the number of cellulitis episodes in lymphoedema [41]. Further, solid evidence supports the efficacy of *prophylactic antibiotics.* Cochrane concludes a reduced incidence of leg cellulitis compared to no treatment/placebo, and is safe [43]. US guidelines suggest initiation after three to four episodes per year [44] while UK recommend considering prophylaxis following two episodes per year [45]. However, the effect stops on discontinuation of prophylaxis [43] and recent UK guidelines on lymphoedema support the use of non-drug measures first (as CDT or treating any dermatitis/wounds), to minimize the use of antibiotics. There is currently no evidence of resistance to penicillin against group A streptococci, in contrast to other antibiotics [45]. Research indicates that patients may be more willing to try preventive strategies with less evidence as daily moisturizing ointments or exercise, compared to prophylactic antibiotics and compression [46].

The frequency of cellulitis on the legs was higher in our previous study [18], with a 1-year incidence of 16% and lifetime-prevalence of 37%. The reasons for the difference is complex, and may include the orthostatic effect and higher presence of bacterial entry sites.

Surprisingly, obesity was not associated with cellulitis in our study, supported by others [20]. Increased BMI is a well-known risk factor for BRCL, increased arm volume [2] and a factor predicting lymphoedema progression after sleeve application [47]. Obesity is also a risk factor for cellulitis of the leg [18, 19]. Further studies are required to understand the mechanisms.

Limitations to our study include the following: Almost all patients had cancer-related lymphoedema, but only 23% of these had a classification of their cancer diagnosis (out of these, 98% had BRCL). The lack of participant characterization may hide important confounders. As 96% of the total cohort were women, we suspect that the majority had BRCL. Secondly, the diagnosis of arm lymphoedema is a topic for discussion, in the absence of international consensus [24, 47]. Thirdly, recruiting mainly from lymphoedema services biases our results towards more severe cases compared to a general health care setting. On the other hand, bias towards more well-treated cases is also likely. Fourthly, “well-treated lymphoedema” was a subjective judgment made by trained investigators. This mirrors the lack of consensus on how to evaluate treatments outcomes of lymphoedema therapy. Lastly, due to the cross-sectional study design conclusions on causation should not be formed.

## Conclusions

Cancer or its treatment is the most frequent cause of lymphoedema of the arm. Cellulitis in lymphoedema is a common international issue, but is also a modifiable risk factor. The risk significantly increases in the advanced stages of lymphoedema. The primary goal of treatment should be to prevent the development of fibrosis and fat accumulation. Achieving good control of the swelling is crucial for cellulitis prophylaxis, associated with an almost 50% lower risk of cellulitis within twelve months.

### Electronic supplementary material

Below is the link to the electronic supplementary material.

Supplementary Material 1

## Data Availability

All data generated or analysed during this study are included in this published article (and its supplementary information files).

## List of Abbreviations

CDT: complete decongestive therapy
BRCL: breast cancer-related lymphoedema
ISL: International Society of Lymphology scale
LIMPRINT: Lymphoedema IMpact and PRevalence- INTernational Lymphoedema Framework

## Footnotes

1 Lymphoedema IMpact and PRevalence- INTernational Lymphoedema Framework.

2 Including overweight.

3 OR cannot be produced as there were no patients with neurological disease in the cellulitis group.
