# Supplementary material for: Factors associated with cellulitis in lymphoedema of the arm – an international cross-sectional study (LIMPRINT)
Source: BMC Infect Dis. 2024 Jan 18;24:102. doi: 10.1186/s12879-023-08839-z (PMC10797905; doi:10.1186/s12879-023-08839-z)
Supplement: Supplementary file 1 — Supplementary Material 1 [file 12879_2023_8839_MOESM1_ESM.docx]

**Appendices**

**Cancer information**

Of the total group the cancer questionnaire was completed in 457 patients of whom 454 had received cancer treatment of whom 395 had no cellulitis and 62 had a recent history of cellulitis.

| **Table A1. Characterization of patients with breast cancer in relation to treatment.** | | |
| --- | --- | --- |
|  | **No cellulitis**  **N (%)** | **Cellulitis**  **N (%)** |
| **N** | **389** | **61** |
| **Lymphoedema after cancer treatment** | |  |
| <3 months | 91 | 6 |
| 3-11 months | 118 | 22 |
| 1-5 years | 137 | 26 |
| 6-9 years | 19 | 4 |
| 10+ years | 16 | 2 |
| **Current cancer status** | |  |
| Cured remission | 117 | 26 |
| Local cancer | 227 | 26 |
| Distant metastasis | 27 | 8 |
| **Treatments** | |  |
| **Surgery** |  |  |
| No | 0 | 0 |
| Yes | 382 | 60 |
| **Radiation** | |  |
| No | 2 | 0 |
| Yes | 308 | 58 |
| **Chemotherapy** | |  |
| No | 5 | 0 |
| Yes | 337 | 57 |
| **Hormone** | |  |
| No | 2 | 2 |
| Yes | 205 | 32 |
| **Molecular target** | |  |
| No | 0 | 1 |
| Yes | 7 | 2 |

| **Table A2. Characterization of surgical treatments in patients with breast cancer.** | | |
| --- | --- | --- |
|  | **No cellulitis**  **N (%)** | **Cellulitis**  **N (%)** |
| **Surgical procedures** | |  |
| 0 | 4 | 0 |
| 1 | 350 | 53 |
| 2 | 31 | 7 |
| 3 | 4 | 1 |
| total | 389 | 61 |
| **Axillary node clearance** | |  |
| No | 2 | 1 |
| Yes | 375 | 60 |
| **Breast reconstruction** | |  |
| No | 3 | 0 |
| Yes | 14 | 2 |
| **Radical mastectomy** | |  |
| No | 3 | 0 |
| Yes | 318 | 53 |
| **Sentinel node biopsy** | |  |
| No | 1 | 0 |
| Yes | 17 | 3 |
| **Simple mastectomy** | |  |
| No | 2 | 0 |
| Yes | 21 | 3 |
| **Wide local excision** | |  |
| No | 0 | 0 |
| Yes | 45 | 6 |
